# Mother-infant interaction context matters for verbal and non-verbal parental mentalization: an initial portrait of associations between parental embodied mentalizing, mind-mindedness, and maternal characteristics in a structured and unstructured context

**DOI:** 10.3389/fpsyg.2023.1176502

**Published:** 2023-07-12

**Authors:** Karine Gagné, Jean-Pascal Lemelin, George Tarabulsy

**Affiliations:** ^1^School of Psychoeducation, University of Montreal, Montreal, QC, Canada; ^2^University Center for Research on Youth and Families (CRUJeF), Quebec, QC, Canada; ^3^Department of Psychoeducation, University of Sherbrooke, Sherbrooke, QC, Canada; ^4^School of Psychology, Laval University, Québec, QC, Canada

**Keywords:** parental mentalization, parental embodied mentalizing, mind-mindedness, cognition and attitudes, psychological characteristics

## Abstract

**Introduction:**

Interest in studying the parental embodied mentalizing (PEM), which refers to implicit and non-verbal processes of parental mentalization, is relatively recent. Therefore, little is known about how PEM, in complementarity with the verbal parental mentalization, is associated with maternal characteristics regarding mother-infant interaction contexts. This exploratory study aimed to investigate the associations between the non-verbal and verbal dimensions of parental mentalization- PEM and mind-mindedness, respectively, - in relation to a wide spectrum of parental characteristics in different interactive mother-infant contexts (toys and no toys).

**Methods:**

Among a sample of 107 mother-infant dyads at moderate psychosocial risk, mothers’ sociodemographic information (age, education, and income), psychological characteristics (depression and anxiety), cognitions (self-efficacy and perceived maternal impact), and attitudes (overprotection and parental warmth) were assessed via self-report questionnaires when the infant was 4 and 8 months old. The PEM and mind-mindedness were evaluated through observation made during a videorecorded sequence of mother-infant interaction in a context of free play with and without toys at 8 months of age.

**Results:**

The results showed distinct associations between PEM and mind-mindedness regarding maternal characteristics: PEM was associated with the mother’s age, education, anxiety and maternal warmth, whereas mind-mindedness was related to cognitions. Both were linked to family income. Regarding mother-infant interaction contexts (toys vs. no toys), the results indicate that the capacity to verbally and non-verbally mentalize differs.

**Discussion:**

These findings shed light on distinctive associations between non-verbal and verbal parental mentalization in relation to certain maternal characteristics, and highlight that the mother-infant interaction context may play an important role in the expression of maternal mentalizing capacity.

## 1. Introduction

In early parent-infant relationships, parental mentalization, which refers to the parents’ capacity to represent and interpret their infant’s behaviors in terms of mental states (e.g., thoughts, emotions), is seen as a key factor associated with the quality of parent-infant interactions (Meins, 1999; Koren-Karie et al., 2002; Slade, 2005; Camoirano, 2017; McMahon and Bernier, 2017; Zeegers et al., 2017). In that context, parental mentalization is understood as a mechanism linking the parents’ mental representations and behaviors toward the infant (Slade, 2005; Meins et al., 2012; Suchman et al., 2012; Koren-Karie and Oppenheim, 2018). By such means, parents with a high capacity for mentalizing, manifested as their capacity to understand the world from the infant’s point of view and to give meaning to the infant’s mental states, would foster a more sensitive parental response to the infant (Meins et al., 2001; Slade, 2005; Koren-Karie and Oppenheim, 2018).

Although previous studies highlight the significant role of parental mentalization in parenting (Camoirano, 2017; McMahon and Bernier, 2017; Zeegers et al., 2017), they mainly examined parental mentalization as a unidimensional construct by focusing on verbal and explicit processes, such as mind-mindedness (Meins, 1999; Meins et al., 2001), parental reflective functioning (PRF; Fonagy et al., 1991; Slade, 2005), and parental insightfulness (Koren-Karie et al., 2002). However, parental mentalization also refers to implicit and non-verbal processes (Shai and Belsky, 2011a,b). As proposed in this study, some authors in the field thus stress the need to study implicit and non-verbal processes of parental mentalization that might complement verbal aspects (Shai and Belsky, 2011a,b; Zeegers et al., 2017; Luyten et al., 2019).

As a means to consider the non-verbal dimension of parental mentalization, Shai (Shai and Belsky, 2011a,b) proposed a new construct, namely parental embodied mentalizing (PEM). PEM refers to parents’ capacity to understand infant mental states (e.g., thoughts, emotions) via movements and bodily gestures, as well as respond on a non-verbal level (Shai and Belsky, 2011a,b). PEM is expressed through the adjustment of parents’ kinesthetic patterns (e.g., quick and jerky vs. slow and gradual rhythm) relating to their infant’s non-verbal signals (Shai and Belsky, 2011a,b; Shai et al., 2017). The term “kinesthetic” refers to the reciprocal dyadic movements between parent and infant, and more specifically to the tempo (fast vs. slow), directionality (shrinking vs. growing), space (near vs. far), tension flow (bound vs. free), pacing (abrupt vs. gradual), and pathways (linear vs. rounded) of the parent-infant interaction.

In contrast to verbal parental mentalization, which rests mainly on the parent’s verbalization (e.g., frequency, coherence, appropriate nature of comments), PEM pertains rather to *how* parents adapt kinesthetically to their infant mental states. For example, if the parent introduces a toy to the infant in a quick, jerky motion and the infant arches their back, does the parent decrease the rhythm of their movements gradually and slowly while moving the toy away from the infant? Here, the focus is on the *way* parents adjust their actions toward their infant, and on their capacity to repair ruptures occurring in interactions, which reflects their understanding of the infant’s mental states and their attempt to connect with those states (Shai and Belsky, 2011a).

The relevance of considering PEM when studying parental mentalization lies in the potential complementarity with the verbal parental mentalization (Shai and Belsky, 2011a,b; Shai et al., 2017; Shai and Meins, 2018; Gagné et al., 2021). Consistently with neuroscience findings indicating that the activation of implicit (non-verbal) or explicit (verbal) mentalization is related to distinct neural circuits (Satpute and Lieberman, 2006; Lieberman, 2007; Luyten and Fonagy, 2015; Luyten et al., 2020), it appears that the verbal (e.g., mind-mindedness, PRF, or parental insightfulness) and non-verbal dimensions (i.e., PEM) of parental mentalization are sometimes activated simultaneously and other times in parallel. In the context of the parent-infant relationship, verbal and explicit parental mentalizing requires a higher level of reflection, cognitive effort, and awareness compared to non-verbal and implicit parental mentalizing, which refers to quicker processes requiring very low levels of cognitive effort and awareness on the parent’s part (Allen et al., 2008; Shai and Belsky, 2011a,b). Hence, the context of interaction may have required a variable state of mind and availability from the parent and may have called on one dimension of parental mentalization more than on the other. In other words, non-verbal mentalization may predominate over verbal mentalization in some contexts, and vice versa. This raises the possibility that the two dimensions have distinct associations and contributions regarding different aspects of parenting and infant development (Shai et al., 2017; Shai and Meins, 2018).

To date, among the four published studies that have examined the association between the verbal and non-verbal dimensions of parental mentalization (Shai et al., 2017; Shai and Meins, 2018; Gagné et al., 2021; Ierardi et al., 2022), three studies supported this hypothesis by showing that PEM was positively and moderately associated with parental mind-mindedness (*r* = 0.25, Gagné et al., 2021; *r* = 0.28, Shai and Meins, 2018) and with PRF (*r* = 0.29; Shai et al., 2017). These results showed the potential complementary contributions of both dimensions of parental mentalization and may provide a better understanding of mentalizing processes underlying in relation to parenthood and the parent-infant relationship.

Beyond the demonstration of associations between the verbal and non-verbal dimensions, the specific study of PEM is relatively recent. More specifically, PEM has been the subject of nine published studies (Shai and Belsky, 2017; Shai and Meins, 2018; Garset-Zamani et al., 2020; Væver et al., 2020; Gagné et al., 2021; Afek et al., 2022; Ierardi et al., 2022; Shai et al., 2022). Most of these studies have focused on the relationships between PEM, maternal sensitivity and infant attachment security (Shai and Belsky, 2017; Shai and Meins, 2018; Væver et al., 2020; Gagné et al., 2021). As expected, these studies suggest a positive association between PEM and maternal sensitivity (Shai and Belsky, 2017; Shai and Meins, 2018; Væver et al., 2020; Gagné et al., 2021), and PEM’s contribution to infant attachment security (Shai and Belsky, 2017; Shai and Meins, 2018; Gagné et al., 2021).

Despite these promising results, very few authors have examined the associations between PEM and individual parental characteristics, such as sociodemographic information, parents’ psychological characteristics, cognitions or attitudes towards their child. Regarding sociodemographic information, studies revealed direct, significant, but weak links between PEM and the mother’s socioeconomic status (Shai and Belsky, 2017; Shai and Meins, 2018), civil status (common law vs. single), age, and education level (Shai and Belsky, 2017). However, Shai et al. (2017) were unable to replicate some of these links, notably those involving the mother’s age and education. It is thus important to continue examining the associations between PEM and sociodemographic characteristics.

Otherwise, one study examined the associations between PEM and parental alliance (i.e., the parent’s perceived cooperation with the other parent) in interactions with infant, demonstrating links positive and moderate links (Shai et al., 2017). Parents’ psychological characteristics in relation to PEM have also been documented in three studies that have examined these links with depression or post-partum depression (Garset-Zamani et al., 2020; Væver et al., 2020; Ierardi et al., 2022). The results showed that mothers who reported post-partum depression symptoms had greater difficulty mentalizing non-verbally (Garset-Zamani et al., 2020; Ierardi et al., 2022). Væver et al. (2020) found no difference between mothers with a depression diagnosis and those without. The only study that had examined the relationship between PEM and maternal anxiety showed that mothers who reported more anxiety symptoms had lower PEM scores (Ierardi et al., 2022). These inconsistent results underline the need to continue investigating the links between PEM and parents’ psychological characteristics, such as depression and anxiety.

In terms of complementarity, available studies tend to support that verbal and non-verbal parental mentalization had differentiated associations according to socioeconomic status, and individual parental characteristics (Shai et al., 2017; Shai and Meins, 2018; Gagné et al., 2021). In Shai and Meins’s study, PEM and mind-mindedness were positively and moderately related to socioeconomic status. However, in Shai et al.’s study, only PRF was associated with maternal education, while maternal age was not correlated with PEM or PFR (Shai et al., 2017). Regarding parental characteristics, PEM and mind-mindedness were both related to maternal sensitivity (Shai and Meins, 2018; Gagné et al., 2021) while PEM was the only dimension associated with parental alliance, post-partum depression or anxiety when PRF was considered (Shai et al., 2017; Ierardi et al., 2022). These results highlight the relevance of considering both dimensions of parental mentalization.

To our knowledge, no other aspect related to individual parental characteristics has been studied in connection with PEM. Considering that parents’ mental predisposition to reflect on their own mental states and those of their infants may affect their capacity to mentalize, it would be relevant to explore associations between PEM and parents’ cognitions and attitudes toward their infant. Furthermore, as mentioned above, further studies investigating the links between PEM and parental sociodemographic and psychological characteristics (i.e., depression, and anxiety) are needed to identify and better understand individual parental characteristics that may affect the parent’s capacity to mentalize non-verbally.

Moreover, adopting a complementarity perspective by studying the verbal and non-verbal parental mentalization would provide a better understanding of the unique role of PEM in parenthood (Shai et al., 2017; Shai and Meins, 2018), as proposed in this study. In line with Shai and Meins’ (2018) recommendations to continue examining the association between mothers’ verbal mentalization and PEM within the same sequence of interactions, we chose to operationalize verbal mentalization through the mind-mindedness measure. Mind-mindedness is based on the parent’s spontaneous verbalizations, which aim to clarify and put into words what the child may be experiencing at the mental level (i.e., emotions, thoughts, desires). Depending on the interactive context and the child’s behaviors, parents’ mind-related comments regarding their child’s mental states are considered appropriate (i.e., accurately reflecting the child’s mental state), or non-attuned (i.e., misinterpreting the child’s mental state).

## 2. The current study

This study aims to explore the associations between non-verbal (i.e., PEM) and verbal (i.e., mind-mindedness) parental mentalization in relation to a wide spectrum of maternal characteristics (sociodemographic, cognitions, attitudes, and psychological characteristics) by considering two observational mother-infant interaction contexts (toys and no toys).

As shown by Madigan et al. (2006), although toys provide some benefits for the observation of parent-infant interactions, this type of context has a structuring effect for the parent–child dyad, thus making certain parental behaviors difficult to observe. Accordingly, the parents’ capacity to mentalize verbally and non-verbally may differ as a function of the interaction context. To our knowledge, only one study has examined verbal parental mentalizing in two different observational contexts (toys and no toys), while suggesting that mind-mindedness tended to differ depending on the interaction context, and moderated the links between maternal mind-mindedness and some aspects of child development (Laranjo et al., 2010). Therefore, through this study, we want to explore if the non-verbal and verbal parental mentalization differ according to a structured (with toys) and unstructured (no toys) context, and in such a case, whether the associations will be distinct regarding maternal characteristics.

The first objective was to examine the links between non-verbal and verbal parental mentalization in relation to sociodemographic characteristics (mother’s age, education, and income), cognitions (self-efficacy, and perceived parental impact), attitudes (overprotection, and parental warmth), and psychological characteristics (depression, and anxiety) regardless of the interaction context. The second objective was to explore if the non-verbal and verbal parental mentalizing scores differ, respectively, according to the interaction context. PEM and mind-mindedness are expected to differ depending on the two observational contexts of mother-infant interaction (toys and no toys). The last objective was to investigate whether the associations between non-verbal and verbal parental mentalization and maternal characteristics were different depending on mother-infant interaction context.

## 3. Method

### 3.1. Participants, procedures, and attrition

This study was part of a larger longitudinal study conducted with mother-infant dyads at moderate psychosocial risk because of their young age (under 25 years), low education level, or low family income. The sample consisted of 107 mother-infant dyads. The mothers were on average 21.80 years old (SD = 1.88, range 15–25). Slightly more than half of them had fewer than 11 years of schooling (55.9%), corresponding to a high school degree. Approximately 36.6% reported having an average family income of under CAN$30,000, the poverty level established by Statistics Canada (2012) in 2009–2010, and 41.6% had an average family income of over CAN$50,000. Most of the mothers were expecting their first child (81.4%). The participants’ civil status was rather homogeneous – 94.1% reported being in a common-law relationship or being married to the biological father. Girls comprised 45.5% of the sample.

Mothers were recruited through a large hospital center in Canada between the years 2008 and 2013. While in their first trimester of pregnancy, the mothers were invited, on a volunteer basis, to take part in the study. Two selection criteria were applied: (1) The mothers had to be under 25 years old at the beginning of the study and (2) Children presenting congenital anomalies, such as cerebral palsy or Down syndrome, were excluded. This research received the required approval from the university research ethics committee (#108.05.11).

The data were collected by two research assistants with prior training in home visits. During the first visit (T1), which took place between the 20th and 24th week of pregnancy, a consent form was read and explained to the mothers, who then signed it. The mothers also filled out various questionnaires concerning their sociodemographic characteristics and their status. The two other home visits (T2 and T3) took place when the infant was 4 and 8 months old. During these visits, the mothers completed questionnaires pertaining to psychological distress experienced and their perceptions of their parental role. At T3, they also participated with their infant in a videorecorded session of free play, which lasted 8 min (with and without toys).

The attrition rate was 8.41% (*n* = 9). The dyads in the initial sample did not differ from those who withdrew, in terms of age (*t* (100) = −1.15, *p* = 0.25), education (*t* (100) = 0.23, *p* = 0.82), income (*t* (99) = 1.10, *p* = 0.28), number of children (*t* (100) = 0.28, *p* = 0.78), civil status (*χ^2^*(1) = 3.45, *p* = 0.18), or the child’s sex (*χ^2^*(1) = 1.35, *p* = 0.25).

### 3.2. Measurements

#### 3.2.1. Maternal embodied mentalizing

PEM was evaluated according to the procedure developed by Shai, which relies on an observation of parent-infant interactions in the context of free play (PEM Coding System Manual – version 2.2, Shai, 2017; Shai and Belsky, 2017). PEM assessment was based on a sequence of parent-infant interactions in the context of free play lasting 8 min (5 min with toys and 3 min without toys), which was videorecorded, when the infant was 8 months old. During the analysis of PEM, the video sound was cut off so the parent’s verbalizations would not influence the evaluation of non-verbal communication. First, embodied circles of communication (ECC), representing non-verbal communicational exchanges between mothers and infants in relation to infant mental states, were identified. Each ECC was classified according to one of five themes and three sub-themes: (a) embodied holding – the parent’s capacity to use their own body to offer a supportive environment for the infant’s mental states; (b) body ownership – the parent’s ability to treat the infant as having its own separate mind and body. This theme is reflected via three sub-themes, such as investigation (i.e., the infant explores the body of its parent by touching the latter’s face), manipulation (i.e., to be playful, the parent uses the infant’s arms to create movements), or stimulation (i.e., the parent tickles or kisses the infant’s body); (c) transitions – when the parent moves the infant’s body around; (d) promoting exploration – when the parent and infant engage in kinesthetic interactions involving a toy; and (e) connectivity – when the parent attempts to connect with the infant’s mental states, for example via a peekaboo game. Afterward, each ECC was evaluated based on the predominant kinesthetic qualities of the parent and infant according to six criteria: directionality (growing and shrinking movement), pacing (velocity of the movements, i.e., abrupt vs. gradual), pathways (linear vs. rounded movement pathways), tension flow (muscular tone involved), tempo (fast vs. slow gestures), and space (near vs. far from the infant’s body center). A global PEM score was assigned on a 7-point scale ranging from 1 (very low) to 7 (very high) reflecting PEM capacities. Based on the two mother-infant interaction contexts (toys and no toys), a PEM score was also assigned on the same 7-point scale. For more details about the procedure, see Shai and Belsky (2017) and Shai and Meins (2018).

The procedure was followed by the first author of the study, who is a certified coder. A random sample corresponding to 20% of the total sample (*n* = 21) was coded by a second certified coder. Intraclass correlations mean varied between *r_i_* = 0.82 and *r_i_* = 0.95 for global PEM, PEM_toys_, and PEM_no toys_, Disagreements were settled by consensus between the two coders.

#### 3.2.2. Maternal mind-mindedness

Maternal mind-mindedness was evaluated with the observational procedure developed by Meins and Fernyhough (2015). Mind-related comments made by parents toward their infant were identified in the same free-play interactions that were used to assess PEM at 8 months. Interactions were transcribed, analyzed, and classified according to the two indicators of mind-mindedness: (1) appropriate mind-related comments (AMRC) and (2) non-attuned mind-related comments (NAMRC).

A comment was qualified as appropriate when (a) the description of the mental states was consistent with the infant’s behaviors; (b) the comment about mental states was referred to a past or future event that was consistent with the context of interaction; (c) the comment helped clarify the child’s mental state; or (d) the mother used the first person to express what the child could say if he or she were able to speak. A comment was considered non-attuned when the mother (a) misinterpreted her infant mental states, (b) proposed another activity when the child was involved in one that he or she was enjoying, or (c) made comments about a past or future event unrelated to the present activity or the child’s current state.

A coder not involved in the coding of other variables and unaware of research hypotheses coded the entire set of data associated with mind-mindedness. Observer XT software was used to enter maternal verbalizations in real time. Global AMRC and NAMRC frequency scores and two specific scores according to the two mother-infant interactions contexts, with and without toys, were calculated. The inter-rater agreement was based on a random sample of 20% mother-infant dyads (*n* = 22). Intraclass correlations varied from *r_i_* = 0.90 (AMRC_global_, AMRC_toys_, AMRC_no toys_) to 0.84 (NAMRC_global_, NAMRC_toys_, NAMRC_no toys_). Disagreements were settled by consensus.

#### 3.2.3. Sociodemographic characteristics

The sociodemographic characteristics were collected via an in-house questionnaire at T1. In this study, the mothers’ age, education, and the average family income were considered variables of interest. Maternal education was evaluated with a Likert scale ranging from 1 (no schooling) to 11 (more than 13 years). The income was established on a Likert scale ranging from 1 (under CAN$10,000) to 8 (over CAN$70,000).

#### 3.2.4. Cognitions and attitudes

The Parental Cognitions and Conduct Toward the Infant Scale (PACOTIS; Boivin et al., 2005) was used to evaluate: (a) *maternal cognitions*, referring to the mothers’ beliefs about their parental role (i.e., parental self-efficacy and perceived parental impact); and (b) *maternal attitudes*, reflecting the mothers’ behavioral tendencies (i.e., parental overprotection, and parental warmth). *Parental self-efficacy* concerns mothers’ perceived ability to perform duties associated with their parental role, particularly in the context where they must provide care to their infant (six items; e.g., “I feel that I am very good at feeding my baby, changing his/her diapers, and giving him/her a bath”). *Perceived parental impact* refers to mothers’ assessment of how their behaviors impact their infant’s development (five items; e.g., “My behavior has a little effect on the development of my baby’s emotions—happiness, fear, anger”). *Parental overprotection* is defined as the adoption of behaviors reflecting excessive concerns about the infant’s safety and protection (five items; e.g., “I insist on keeping my baby close to me at all times, within my eyesight, and in the same room as I am”). *Parental warmth* refers to the sense of pleasure and affection that mothers feel and demonstrate when interacting with their infant (nine items; e.g., “I feel so happy and so mellow when my child smiles at me”). Based on an 11-point Likert scale (1 = “not at all what you think or do” to 11 = “exactly what you think or do”), mothers had to indicate if it was an appropriate description of their actions, thoughts, or emotions when they were interacting with their infant. Given that the scores at ages 4 and 8 months were strongly correlated (*r* = 0.60 self-efficacy, *r* = 0.45 perceived parental impact, *r* = 0.66 overprotection, and *r* = 0.72 maternal warmth), a mean score for the two measurement times was calculated and used in the analyses to reflect the mothers’ perceptions and general behavioral tendencies toward their infant’s behaviors.

A high score indicated that mothers’ perceptions and behavioral tendencies toward their infant were high. In this study, the scale’s internal consistency was satisfactory: 0.79 (Cronbach’s α self-efficacy), 0.69 (α perceived parental impact), 0.68 (α overprotection), and 0.79 (α parental warmth).

#### 3.2.5. Psychological characteristics

Maternal psychological characteristics were evaluated using the Symptom Checklist-90-Revised, which was designed to assess psychological distress (SCL-90-R; Derogatis, 1994). Two scales from the SCL-90-R were retained: depression (13 items; e.g., “feeling lonely or feeling blue”), and anxiety (10 items; e.g., “nervousness or shakiness inside”). Mothers had to indicate, on a 5-point Likert-type scale ranging from (0) not at all to (4) extremely, if the symptom was present in the past seven days when the infant was 4 and 8 months old. As the scores at 4 and 8 months were strongly correlated (*r* = 0.66 depression, *r* = 0.81 anxiety), a mean score for the two measurement times was calculated and used in the analyses to reflect the mother’s general psychological state.

A high mean score meant the psychological symptoms were severe. As recommended by Gadermann et al. (2012) for scales that have less than 7 points, we used polychoric alphas to evaluate the scales’ internal consistency. In the current study, the psychometric qualities indicated excellent internal consistency for the depression (0.92) and anxiety (0.94) scales.

## 4. Data analyses

First, the normal distribution of each variable was verified by examining the kurtosis and skewness, and descriptive analyses were conducted. Based on the normal theory estimation which postulates that *p*-values will be respectably accurate when data are skewed less than 2.0 and the kurtosis is less than 9.0 (Gignac, 2019), the distribution of each variable in this study was examined and showed a normal distribution. Table 1 presents the descriptive statistics of PEM, mind-mindedness, cognitions, attitudes, and psychological characteristics. Second, to examine the associations between PEM and mind-mindedness (two indicators, AMRC and NAMRC) in relation to sociodemographic characteristics, cognitions, attitudes, and psychological characteristics, correlation analyses were performed by considering or not the two interaction contexts (Objective 1 and 3). *T*-tests were also conducted to explore if PEM and mind-mindedness scores differed, respectively, according to the interaction context (Objective 2).

**Table 1:** Descriptive statistics of PEM, mind-mindedness, and maternal characteristics.

Variables^a^	Mean	SD^f^	Range
NVPM – PEM^b^			
PEM_global_	3.54	0.58	1.95–4.94
PEM_toys_	3.74	0.61	2.07–5.00
PEM_no toys_	3.41	0.65	1.59–4.92
VPM – Mind-mindedness^c^			
AMRC^d^_global_	9.32	5.07	0–22
AMRC_toys_	5.65	3.38	0–15
AMRC_no toys_	1.60	1.91	0–12
NAMRC^e^_global_	3.19	2.96	0–12
NAMRC_toys_	3.92	2.86	0–13
NAMRC_no toys_	1.65	1.91	0–8
Cognitions			
Self-efficacy	9.54	0.88	6.75–11.00
Perceived parental impact	9.82	1.25	5.60–11.00
Attitudes			
Overprotection	5.13	1.80	1.20–10.30
Parental warmth	9.75	0.95	6.00–11.00
Psychological characteristics			
Depression	0.60	0.42	0.04–2.08
Anxiety	0.35	0.48	0.00–2.50

a Sociodemographics information (i.e., mother’s age, education, and income) were reported in the section “participants, procedure, and attrition”.

b Non-verbal parental mentalization – parental embodied mentalizing (PEM).

c Verbal parental mentalization – mind-mindedness.

d AMRC, Appropriate mind-related comments.

e NAMRC, Non-attuned mind-related comments.

f SD, standard deviations.

## 5. Results

In the global model including the two mother-infant interactions contexts, PEM was positively associated with the mother’s age, education, income, and maternal warmth (see Table 2), whereas AMRC scores were positively related to the perceived maternal impact (cognition). NAMRC was only linked negatively to income.

**Table 2:** Associations between PEM, mind-mindedness, and maternal characteristics according to the mother-infant interaction context.

	Global			With toys			Without toys		
	PEM^a^	AMRC^b^	NAMRC^c^	PEM	AMRC	NAMRC	PEM	AMRC	NAMRC
Sociodemographics									
Mother’s age	**0.32** ^**^	−0.05	−0.07	**0.36** ^**^	−0.07	−0.04	**0.20** ^**†**^	0.04	−0.07
Education	**0.37** ^**^	0.01	0.11	**0.43** ^**^	0.01	0.11	**0.32** ^**^	0.01	0.06
Family income	**0.29** ^**^	0.06	**−0.22** ^*^	**0.37** ^**^	0.06	−0.13	**0.19** ^**†**^	0.05	**−0.22** ^*^
Cognitions									
Self-efficacy	0.14	−0.09	−0.05	0.12	**−0.22** ^*^	−0.02	0.04	0.09	−0.06
Perceived parental impact	0.11	**0.28** ^**^	0.09	0.14	**0.24** ^*^	0.06	0.05	**0.19** ^**†**^	0.07
Attitudes									
Overprotection	0.02	−0.12	−0.12	−0.03	**−0.23** ^*^	−0.06	0.02	0.03	**−0.17** ^**†**^
Parental warmth	**0.24** ^*^	−0.08	0.02	**0.23** ^*^	−0.12	0.04	0.15	0.02	−0.03
Psychological characteristics									
Depression	0.00	0.06	−0.10	−0.09	−0.08	−0.07	0.11	**0.21** ^*^	−0.09
Anxiety	−0.13	0.05	−0.06	**−0.21** ^*^	−0.11	−0.11	−0.02	**0.21** ^*^	0.02

^**^p < 0.001; ^*^p < 0.05; ^†^*p* < 0.10; ^a^PEM, Parental embodied mentalizing; ^b^AMRC, Appropriate mind-related comments; ^c^NAMRC, Non-attuned mind-related comments.

Regarding the context with toys and without toys and maternal mentalizing capacity, *t*-tests demonstrated that the mean scores for PEM (*t* (97) = 6.91, *p <* 0.001) and AMRC (*t* (103) = 5.02, *p* < 0.001) differed depending on the interaction context. The mothers’ capacity to non-verbally and verbally mentalize was higher in a structured context involving toys. No significant difference was observed for NAMRC (*t* (103) = −0.72, *p* = 0.48).

PEM_toys_ was associated with the same maternal characteristics as in the global model, where a link with maternal anxiety was found (see Table 2). As in the global model, AMRC_toys_ was associated with perceived parental impact in addition to being related to self-efficacy and to overprotection. No significant association was observed with NAMRC_no toys_ scores and maternal characteristics.

In the sequence without toys, PEM_no toys_ was significantly associated with the mothers’ education and tended to be related to the mothers’ age or income. AMRC_no toys_ was associated with depression and anxiety, and was marginally related to perceived parental impact. As in the global model, NAMRC_no toys_ was negatively correlated with income, and in that specific context tended to be linked to overprotection.

## 6. Discussion

The purpose of this exploratory study was to examine associations between non-verbal (PEM) and verbal (mind-mindedness) parental mentalization in relation to a wide spectrum of maternal characteristics (i.e., sociodemographic, cognitions, attitudes, and psychological characteristics) by considering or not two mother-infant interaction contexts (toys or no toys). Moreover, this study aimed to explore if the non-verbal and verbal parental mentalizing capacity differed according to an interaction context with toys or without toys.

Regardless of the interaction context, this study suggests that maternal cognitions are related to the mother’s verbalizations (i.e., appropriate mind-related comments) while mother’s attitudes are associated with behavior (PEM). Specifically, a higher PEM was associated with greater maternal warmth, while mothers who reported better perceptions of their parental impact on their infant commented more appropriately on their infant’s mental states. This therefore might indicate that what is going on in the parent’s head would be translated into verbal mentalizing comments – what the parent will say to their infant – while non-verbal mentalization would be more reflected in the parent’s behaviors or attitudes, which is consistent with Shai and Belsky’ (2011a,b) theoretical ideas indicating that PEM is especially significant regarding the parent’s ability to repair dyadic miscoordination. Moreover, in line with Shai and Meins (2018), who point out that these two dimensions of parental mentalization seem to differ conceptually and in terms of neural activation, our results tend to corroborate this idea by highlighting that the mechanisms underlying the non-verbal and verbal processes of parental mentalization appear related to distinct parental patterns.

In addition, by documenting the associations between PEM and sociodemographic characteristics (i.e., mothers’ age, education, and income), this study helps to identify the maternal characteristics related to non-verbal parental mentalization in at-risk psychosocial contexts. Our results suggest that within a sample of mother-infant dyads at moderate psychosocial risk, mothers are more likely to present low PEM if they are younger, less educated, and have low income. Although our results are in the expected direction and consistent with those found in previous studies, it appears that associations between PEM and mother’s age or education were generally stronger in this study than those reported by Shai et al. (see Shai et al., 2017; Shai and Belsky, 2017; Shai and Meins, 2018). The fact that our study was conducted exclusively with mother-infant dyads at moderate psychosocial risk, which was not the case with samples from previous studies, may explain the stronger correlations observed in this study. Therefore, in line with existing evidence showing that a low socioeconomic status is associated with greater parental developmental issues (NICHD Early Child Care Research Network, 2005; Stack et al., 2012; Kolomeyer et al., 2016; Ensink et al., 2017), it can be argued that younger mothers with less education are more inclined to present difficulties in conceiving, interpreting, and demonstrating their appreciation of their child’s mental states non-verbally. Considering the scarce studies on PEM (Shai et al., 2017; Shai and Belsky, 2017; Shai and Meins, 2018), this study sheds light on the main sociodemographic characteristics most likely to affect PEM, and which should be considered in future mentalization-based interventions with psychosocially at-risk mother-infant dyads.

In accordance with our hypothesis, we found that non-verbal and verbal mentalization capacity differed according to the context: Mothers showed higher PEM and appropriate mind-related ability in a structured context with toys compared to an unstructured context without toys. Consistently with theorists supporting that mentalization is more challenging when emotions involve feeling intense and stressful (Fonagy et al., 2002), our study suggests that the dyadic interaction without toys seems to be more stressful, which could explain why the corresponding PEM and appropriate mind-related scores were significantly lower. The lack of difference for the non-attuned mind-related verbalizations (NAMRC) between the two contexts could be explained by the social desirability effect. This raises the possibility that in the contexts where mothers are filmed, they might have made less negative mind-related comments, which is consistent with previous studies indicating few associations between non-attuned mind-related comments and parenting behaviors (McMahon and Bernier, 2017). Despite this possibility, this study highlights that in a day-to-day parent-infant interaction, the parents’ capacity to mentalize verbally and non-verbally is flexible, and adjustable according to the nature of the context. With a view to obtaining a better portrait of this parental capacity, it is thus important to assess parents’ mentalization based on different observational contexts of parent-infant relationships.

When the context in which the action of verbal and non-verbal mentalizing takes place is considered in relation to maternal characteristics, the portrait tends to differ between interactions involving toys and those without toys. In a context with toys, a higher PEM was associated with less anxiety symptoms and greater parental warmth toward the infant, whereas more appropriate mind-related comments were related to a better perceived parental impact, lower self-efficacy, and a less overprotective attitude. In the interactions without toys, the portrait differs in showing that PEM was associated only with education level, and appropriate mind-related comments was linked only with maternal psychological states. These results imply that interactions structured by a toy may facilitate positive exchanges and bidirectional communication between parent and infant, which could explain why more associations between verbal and non-verbal parental mentalization according to maternal characteristics were observed in a context with toys. As mentioned previously, interaction without toys appears more stressful, which could lead to mentalizing impairments, such as hyper-mentalization. In this perspective, the positive link between appropriate mind-related comments and anxio-depressive symptoms observed only in the interaction without toys could be explained by the presence of hyper-mentalization (Luyten et al., 2017). According to Fonagy et al. (2016), in some high-risk samples, the parent may be more inclined to use mental language that reflects hyper-mentalization rather than a truly high level of verbal parental mentalization, which might be the case in this study.

By exploring the associations between PEM and a wide spectrum of maternal characteristics, this study provides a first portrait of the main potential maternal determinants involved in non-verbal mentalizing capacity. This study’s consideration of the verbal (mind-mindedness) and non-verbal (PEM) parental mentalization also helps identify the specific associations between these two dimensions according to different maternal characteristics. Our study thus provides insight into the unique role of PEM and its potential distinctive contribution to different facets of parenting, and parent-infant relationships.

### 6.1. Limitations and futures directions

This study has some limitations that must be considered in interpretating its results. In this study, the results were based on mothers’ perceptions of their psychological state, cognitions and parenting attitudes towards their baby, as revealed by the self-report questionnaire. One of the risks associated with this type of assessment is that parents may overestimate their parenting abilities, or conversely underestimate the behavioral or psychological difficulties they encounter. Thus, some parents may have underestimated their difficulties, either out of social desirability, or because they were less inclined to recognize the most problematic symptoms or behaviors. Secondly, the sample consists exclusively of mother-infant dyads at moderate psychosocial risk, which limits generalization to father-infant dyads or to populations not at risk. The at-risk nature of this sample also represents a strength, in that to our knowledge, previous studies concerning PEM included mother-infant dyads who were not at risk.

Given that this study is, to our knowledge, the first one exploring the associations between PEM and a wide spectrum of maternal characteristics in a context of psychosocial risk, our results should be replicated. In this regard, future research should continue to examine the associations between PEM and verbal parental mentalization in relation to different aspects of parenthood and infant development. Furthermore, as several other researchers in the field have highlighted, studies conducted with father-infant dyads are needed to better understand the differences and similarities in mentalization between mothers and fathers (McMahon and Bernier, 2017; Shai and Meins, 2018). This recommendation is even more relevant for PEM because there is only one published study where PEM has been evaluated with father-infant dyads.

## 7. Conclusions and implications

By adopting a complementary perspective of non-verbal and verbal parental mentalization, this study contributes to a better understanding of the unique role of PEM in parenthood. This study extends the current knowledge about PEM, parental mentalization, and parenting by highlighting: (1) differential associations between the verbal and non-verbal parental mentalization in relation to a wide spectrum of maternal characteristics and (2) the significant role played by the context of mother-infant interactions in the expression of a mother’s capacity to mentalize verbally and non-verbally.

This initial portrait raises awareness of the need to integrate non-verbal parental mentalization more systematically when studying parental mentalization. Moreover, our results highlight the relevance of considering both verbal and non-verbal in parental mentalization-based interventions. To date, several interventions targeting parents’ capacity for mentalization in the context of psychosocial risk have been developed and implemented; for example, Minding the Baby (Slade et al., 2005, 2020) or Mothering Inside Out (Suchman et al., 2008, 2018). In a perspective of complementarity, it appears relevant to include PEM in such interventions.

## Data availability statement

The datasets presented in this article are not readily available because the participants of this study did not give written consent for their data to be shared publicly. Requests to access the datasets should be directed to the corresponding author, Karine Gagné.

## Ethics statement

The studies involving human participants were reviewed and approved by University research ethics committee (#108.05.11). Written informed consent to participate in this study was provided by the participants’ legal guardian/next of kin.

## Author contributions

KG: conceptualization, methodology, validation, formal analysis, data curation, visualization, writing original draft, and writing review. J-PL: methodology, validation, resources, writing-review and editing, supervision, project administration, and funding acquisition. GT: methodology, investigation, validation, resources, writing-review and editing, supervision, project administration, and funding acquisition. All authors contributed to the article and approved the submitted version.

## Funding

This study was funded by grants to J-PL and GT from the Social Sciences and Humanities Research Council of Canada (SSHRC, grants 410-2010-1950 and 410-2006-2431) and the Fonds de recherche du Québec - Société et culture (FRQSC, grant 2010-NP-131273). KG was awarded a Doctoral Research Scholarship from the SSHRC, the FRQSC, and the University Center for Research on Youth and Families (CRUJeF).

## Conflict of interest

The authors declare that the research was conducted in the absence of any commercial or financial relationships that could be construed as a potential conflict of interest.

## Publisher’s note

All claims expressed in this article are solely those of the authors and do not necessarily represent those of their affiliated organizations, or those of the publisher, the editors and the reviewers. Any product that may be evaluated in this article, or claim that may be made by its manufacturer, is not guaranteed or endorsed by the publisher.
